# Towards successful digital transformation through co-creation: a longitudinal study of a four-year implementation of digital monitoring technology in residential care for persons with dementia

**DOI:** 10.1186/s12913-019-4191-1

**Published:** 2019-06-10

**Authors:** Janne Dugstad, Tom Eide, Etty R. Nilsen, Hilde Eide

**Affiliations:** 1 The Science Centre Health and Technology, Faculty of Health and Social Sciences, University of South-Eastern Norway, Drammen, Norway; 2 The Science Centre Health and Technology, School of Business, University of South-Eastern Norway, Drammen, Norway

**Keywords:** Service design, Absorptive capacity, Risk, Triple helix, Workflow, Radical innovation

## Abstract

**Background:**

Implementation of digital monitoring technology systems is considered beneficial for increasing the safety and quality of care for residents in nursing homes and simultaneously improving care providers’ workflow. Co-creation is a suitable approach for developing and implementing digital technologies and transforming the service accordingly. This study aimed to identify the facilitators and barriers for implementation of digital monitoring technology in residential care for persons with dementia and wandering behaviour, and explore co-creation as an implementation strategy and practice.

**Methods:**

In this longitudinal case study, we observed and elicited the experiences of care providers and healthcare managers in eight nursing homes, in addition to those of the information technology (IT) support services and technology vendors, during a four-year implementation process. We were guided by theories on innovation, implementation and learning, as well as co-creation and design. The data were analysed deductively using a determinants of innovation framework, followed by an inductive content analysis of interview and observation data.

**Results:**

The implementation represented radical innovation and required far more resources than the incremental changes anticipated by the participants. Five categories of facilitators and barriers were identified, including several subcategories for each category: 1) Pre-implementation preparations; 2) Implementation strategy; 3) Technology stability and usability; 4) Building competence and organisational learning; and 5) Service transformation and quality management. The combination of IT infrastructure instability and the reluctance of the IT support service to contribute in co-creating value with the healthcare services was the most persistent barrier. Overall, the co-creation methodology was the most prominent facilitator, resulting in a safer night monitoring service.

**Conclusion:**

Successful implementation of novel digital monitoring technologies in the care service is a complex and time-consuming process and even more so when the technology allows care providers to radically transform clinical practices at the point of care, which offers new affordances in the co-creation of value with their residents. From a long-term perspective, the digital transformation of municipal healthcare services requires more advanced IT competence to be integrated directly into the management and provision of healthcare and value co-creation with service users and their relatives.

## Background

Digital monitoring has become an increasingly important application among the health information technologies (IT) in long-term care, such as residential care facilities for persons with dementia [1, 2]. Implementation of monitoring technologies potentially reduces staff burdens and enhances safety, increases resident freedom and prevents elopements and wandering behaviour in persons with dementia [3–9]. This includes persons referred to as night wanderers. Sleep disturbances and wandering upon awakenings in combination with night-time agitation pose severe challenges in caring for these persons [10–12].

The research literature provides recommendations as to how implementation of monitoring technologies can be facilitated (e.g. [4, 13, 14]). However, many healthcare professionals (HCPs) and service organisations are reluctant to introduce such technologies [2, 15]. There are multiple causes for this reluctance, including ethical considerations, fear that technology will cause attenuation of the care relationship, lack of appropriate infrastructure, and a general lack of knowledge and skills in relation to digital health [3, 16–21]. In a recent literature review, Granja et al. [22] found quality of healthcare to be the major facilitator and shortage of finance the major barrier in the implementation of eHealth interventions, including monitoring technologies. The intervention’s influence on existing workflow was the single most important factor to predict success or failure. There is a need for further identification of facilitators and barriers to ensure that all factors are considered when defining the development and implementation strategy of specific eHealth interventions [22].

Intelligent assistive technologies (IATs) [23] are monitoring technologies with computation capability and the ability to communicate information through a network. These are complex technologies that require new skill sets and perspectives, and their development must be responsive to the needs of their users and simultaneously be commercially viable [24]. A high number of the more recently introduced IATs lack clinical validation; i.e. technical feasibility and usability have predominately been tested through simulations [15]. Therefore, study designs involving multiple stakeholders in technology development processes are recommended [4, 16, 24, 25]. New technologies transform services, including contexts, service provision and experiences with respect to organisations, employees and users. Therefore, there is a need for research into service innovation by leveraging service design and understanding value creation in this context [26]. These are time-consuming processes, but most implementation studies report retrospectively from early phases and there are few innovations studies in the field that cover long periods of time [27].

The current article is a longitudinal case study of the implementation of digital monitoring technology over a four-year period. The article explores the barriers and facilitators during the implementation and the strategic role of co-creation processes to overcome resistance, improve functionality and ensure quality of care. In a previous article from the first year of these processes, four main forms of resistance to the implementation were identified: i.e. organisational, cultural, technological and ethical resistance [20]. Resistance was triggered by perceived threats to stability and predictability, role and group identities and basic healthcare values.

## Conceptual framework: innovation and implementation through co-creation

Innovation in health service delivery and organisation has been defined as “a novel set of behaviors, routines, and ways of working that are directed at improving health outcomes, administrative efficiency, cost effectiveness, or users’ experience and that are implemented by planned and coordinated actions” [28]. This definition captures many aspects of the innovation processes under study, as novel technologies and new ways of working were developed and implemented to benefit service users and healthcare organisations. Service innovations are usually categorised according to the degree of change, type of change, novelty and means of provision [29]. Most innovations in the public sector are incremental, but still disruptive, i.e. they are changes that potentially cause improvement [27, 30]. Radical innovations usually refer to products, such as breakthrough technologies, that their intended users perceive as novel, disruptive and hard to adopt, disturbing prevailing habits and behaviour [31]. Radical innovations rely on a series of incremental innovations to be fitted into a system or context in a form that is acceptable to the intended users [32]. Regarding the type of change, discussions of product and process innovations are predominant in the literature. Other types include position, strategic, governance and rhetorical innovation [30].

The innovation process is traditionally described by the stages of dissemination, adoption, implementation and continuation [33]. The transformative nature underpins the need for learning and development of new knowledge as an organisation implements an innovation e.g. [27, 28]. The more radical the innovation, the more necessary it is to teach the users how to adopt and use it [34]. The organisation’s absorptive capacity includes capabilities for problem solving and learning new knowledge generated externally, as well as technological infrastructure, leadership, internal knowledge sharing and relational capability [35–37]. The absorptive capacity builds cumulatively on the existing base of skills and knowledge [27, 35], including tacit knowledge [38]. Absorptive capacity is an antecedent and strong predictor for innovation and knowledge transfer [28, 37].

Implementation of innovative technology within complex organisational systems, such as healthcare, involves various cycles of iteration as technological, social and organisational dimensions gradually align (or not) over time [18]. Interacting influences known as determinants of innovations [39] and determinants of healthcare professional practice [40] contribute to the multidimensionality of the innovation process, and enable or prevent the improvement, or change, in the specific context or practice. Information about such barriers and facilitators is useful for controlling the implementation strategy and a determinant of innovation framework helps to focus this study on the essential processes of behavioural change, which are complex in clinical settings [41, 42].

The triple-helix model [43] is an innovation strategy where public sector organisations, private sector companies and academia collaborate and co-create. This strategy allows its intended users to be involved in design and development processes of products, processes and services, and involvement is likely to improve adoption and post-implementation satisfaction [28, 44]. Resources can be accessed from other actors through absorption, acquisition, sharing and resource co-creation [45].

Co-creation is an interaction where actors jointly produce a mutually valued outcome based on assessments of the risks and benefits of proposed courses of action and decisions based on dialogue, access to information and transparency [46]. Cutting through a broad variety of concepts and theories regarding co-creation [47–52], its central elements include defining and creating value through iterative processes including value propositions, resource integration and learning processes. Public sector services are suitable for co-creation because they are discreet and intangible, focusing on the users consuming the service as it is produced or delivered [53]. Traditionally, the value-creation process is said to occur as the user consumes or uses a product or service [54]. According to Oertzen et al. [49], co-creation in services “manifests itself in different forms depending on the phases of the service process (co-ideation, co-valuation, co-design, co-test, co-launch, co-production and co-consumption) and is influenced by a contextual, multi-actor network”. Co-creation includes creative collaboration connected to design processes as well as the usage or delivery of a service [55]. Actively involving intended users through participatory design processes has traditionally been emphasised in IT design [56]. Service design processes aim to develop services that are useful, usable and desirable from the service users’ perspective [57]. Service design applies to all parts of a service, including planning and organising people, infrastructure, communication and material components [55].

## Methods

### Aim and study design

The overall aims of this article are a) to identify facilitators and barriers, and b) to explore co-creation practices as an innovation strategy during four years of implementation of a digital monitoring technology in long-term residential care for persons with dementia who were night wanderers. The study had a longitudinal case study design [58] with elements of transformational action research [59]. Action research elements included researcher participation in the project design and planning activities, participation in and facilitation of knowledge-sharing and reflection processes during workshops and meetings, and presentation of preliminary research findings to the steering group and during workshops, which informed the iterative innovation activities.

### The case: a digital night surveillance intervention

The present study is based on the Digital Night Surveillance Innovation Project, which was a combined innovation and research project initiated by a triple-helix-inspired network that developed digital technologies for municipal healthcare services. Between 2009 and 2012, vendors from a small-sized enterprise had developed a distributed IAT system, i.e. the digital monitoring technology system used in this study, which potentially offered increased safety for persons with dementia who were night wanderers. To access the immature market of municipal healthcare organisations, the vendors organised a project for implementation utilising public sector-sponsored incentive programs to minimise economic risk for the municipalities. Based on their access to funding, the vendors and three municipalities initiated the implementation in 2013, and successively recruited more partners and established a formal consortium of eight municipalities and two technology companies for the main project from 2014 to 2017. A group of nine researchers from two universities, including the authors of this paper, participated in the consortium.

The implementation strategy encompassed a variety of co-creation activities combining human-centred and service designs, as well as participatory design methodologies. Workshops constituted a major arena for co-creation during the implementation, as detailed below. An orchestrator managed the implementation project in co-operation with a project group comprised of the local project managers and vendors. Within the municipalities, local politicians made formal decisions to enter the project based on preparations by municipal top management. The organisational units of adoption, one per municipality, were dementia care wards in nursing homes within the municipal healthcare organisations. Healthcare workers and registered nurses, i.e. care providers, working on the night shift were anticipated to be the main group of users to adopt the monitoring technology.

Sixty-seven installations of the monitoring technology system were implemented. The system consisted of an Internet-based portal built on a platform solution that included novel Internet-of-Things (IOT) middleware, which could handle and unify data from multiple hardware protocols and allowed integration of e.g. bed-exit or door sensors from different manufacturers. Thus, the system offered a unique feature where the care providers could operate multiple technologies simultaneously via a personal computer (PC), tablet or smartphone of their choice. A short message service (SMS)-mediated alarm alerted care providers when predefined scenarios occurred, such as a resident leaving their bed. The portal allowed adjustments of settings at any time to match the needs, behaviour and progression of dementia of individual residents, including the sequence and timing of input from a variety of sensors. No other monitoring technology systems available on the market at the time offered these affordances. Upon installation, the final stages of designing user interfaces on the applications and operating systems chosen by each municipality, as well as integration of suitable sensor technology, would take place. The monitoring technology was in compliance with regulations of data protection and privacy, as well as the legal framework for monitoring persons with dementia using sensors. According to the Norwegian Patients Rights’ Act, municipal health and care services may decide on the use of technology for notification and localization as part of services to patients over the age of 18 who do not have capacity to consent. The measure must prevent or limit the risk of injury to the patient, be in reasonable proportion to the relevant risk and appear to be the least invasive option. It should be likely that the patient would have given permission for the measure. The provision does not apply if the patient opposes the measure.

### Participants and data

Data collection took place between June 2013 and September 2018. The data included 23 interviews, strategic documents, participatory observations and process data from seven workshops, as well as observations of local training sessions and numerous meetings. The meetings were steering group meetings, project group meetings, local staff meetings, information meetings for residents and relatives, meetings between vendors and single municipalities, and meetings between IT and healthcare services. Data was not collected in care settings, and not from residents or relatives. All participants in research settings consented to participation in the research study. The study complied with the tenets of the Declaration of Helsinki.

#### Workshops

Data from workshops were collected between November 2014 and September 2016. The workshops (not including the final dissemination seminar) were attended by participants (*n* = 172) from municipal healthcare service staff (*n* = 89) and IT service staff (n = 8), vendors (*n* = 30), research institutions (*n* = 14), non-governmental organisations (n = 3), other public sector organisations (*n* = 5), innovation and funding agencies (*n* = 20) and external experts (n = 3).

The orchestrator and researchers facilitated workshops, where the researchers and other experts initially would introduce a theme predetermined by the project group. Then all participants engaged in co-creational activities related to the theme and thus contributed to the progress of the implementation. The researchers documented the results of such activities and made them available to the participants in a reasonable time. In addition, the vendors and the local project managers presented updates during workshops and the researchers presented preliminary research results. There were opportunities for generating and prioritising ideas, discussions and exchanges of experiences. The workshops usually lasted for two days, from lunch to lunch, with a social event during the evening. Workshop locations were close to the participating municipalities and one took place in Sweden in co-operation with a corresponding triple-helix network.

#### Interviews

The sample consisted of 21 individual interviews (*n* = 16) and two focus group interviews, i.e. one with HCPs (*n* = 9) and one with the vendors (*n* = 4) (Table 1). Fifteen interviews were performed between August 2013 and April 2016, and informants were interviewed up to three times. Individual interviews were performed at a place of the informants’ choice, normally at their workplace. The focus group interviews took place in co-creation activity settings. The interviews started with a “grand tour” question (around the table if in a focus group) to elicit the informants’ perception of the implementation and their own participation in the project. Two main topics were then discussed with the informants: i.e. if any need for new competence had emerged and how it had been dealt with; and if there had been changes to the job situation or organisation of HCPs. The interviews were semi-structured, recorded and transcribed verbatim. Please c.f. Nilsen et al. [20] for the interview guide. Purposeful selection assured inclusion of informants representing the enterprises (*n* = 4) as well as the initial three municipalities (n_1_ = 6, n_2_ = 5 and n_3_ = 6) that participated throughout the entire project period from 2013 to 2017. As a validation of information regarding the municipal planning and preparation process, JD interviewed the orchestrator, three local project managers and two vendors over the phone in April–September 2018. These interviews lasted for 10 to 45 min and were documented by notes.

**Table 1:** Overview of interviews and informant characteristics

Informant	PM	Manager	Superuser	Nightshift	Profession	II	FG
1	x	x			RN	2	
2	x				RN	2	
3	x				RN	2	
4		x			RN	1	1
5		x			RN	2	
6		x			RN	1	1
7	x		x		RN	2	1
8			x	x	RN		1
9			x	x	RN	1	
10			x	x	HCW	1	1
11			x	x	HCW	1	1
12				x	HCW	1	
13				x	RN		1
14				x	RN	1	
15				x	RN		1
16				x	RN		1
17					T	1	1
18					T	1	1
19					T		1
20					T		1
21					IT	1	
22					O	1	

Abbreviations: *FG* Focus Group Interview, *HCW* Healthcare Worker, *II* Individual Interviews, *ITM* Information Technology Manager, *O* Orchestrator, *PM* Project Manager, *RN* Registered Nurse, *T* Technologist

#### Data analyses

Data from qualitative interviews were analysed by content analysis [60], followed by an inductive phenomenological hermeneutical analysis inspired by Lindseth and Norberg [61]. The first step consisted of deductive qualitative analysis and mapping of the transcribed interviews [60] against the constructs in the measurement instrument for determinants of innovation (MIDI) framework [62]. JD and HE did the analysis. The MIDI framework [62] encompasses the innovation process and strategy, and captures four broad categories of essential determinants, as evaluated by healthcare professionals, who are considered to be the adopting users during the implementation of innovations in larger healthcare organisations. The category associated with the innovation includes determinants such as correctness, complexity and compatibility. The adopting user category encompasses benefits, professional obligations, knowledge and perceived satisfaction of patients. The category for organisational attributes includes determinants such as management involvement, staff capacity, resources, and information and performance feedback. Legislation and regulations constitute the final socio-political category.

The second step consisted of an inductive analysis of the same material by putting the MIDI framework in parenthesis to grasp the essence of the meanings of the informants’ expressions of their experiences of the innovation processes. JD and TE performed several iterations of the inductive exploration, the latter without any knowledge of the results of step 1. The aim was to group facilitators and barriers into themes. JD performed the initial inductive coding and then condensed the data excerpts. The data were complex; therefore, physical organising and structuring was needed. Thus, the data excerpts were printed and cut into separate units. JD and TE sorted and reorganised the data and this analysis resulted in the main structure of themes.

In the third and final step, observational data, process data and strategic documents were examined to enrich the exploration of processes that were found to be essential during implementation. Therefore, the data from interviews, observations and text analysis were integrated. Utilising a phenomenological hermeneutical approach [61], JD and TE interpreted the data excerpts in an iterative manner by reading and critiquing each other’s texts. We abstracted the data to form subcategories in the form of facilitators and barriers. The subcategories were then further condensed into categories and reviewed in a timeline perspective. JD, TE, HE and EN contributed in finalising the themes, categories and timeline.

Threats to validity were met by co-operating within the research team in all phases of the research project, which ensured open discussion and deep knowledge of the context. The reliability of the study was strengthened through researcher triangulation. A further layer of discussion and reflexivity about the data and their interpretation with consortium members complemented the interdisciplinary reflections and discussions. Detailed descriptions of the research approach were included to meet threats to reliability.

## Results

**Fig. 1:**
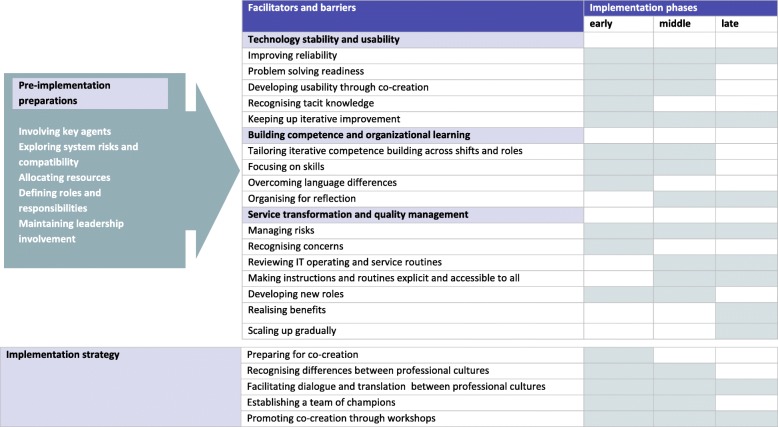
The Lifespan of the Implementation of Digital Monitoring Technology

Through the deductive analysis of the interviews, we were able to identify all of the determinants of the MIDI framework. The analysis provided deeper insights, but the MIDI framework did not cover the entire material. In the following, the results of the inductive analysis (step 2 and 3) will be presented. Five major categories of barriers and facilitators of innovation were identified, including subcategories for each category: i.e. factors, processes and actions that proved to facilitate the implementation when completed or impede the implementation when not. The presentation of categories and subcategories include descriptions of how facilitators and barriers were experienced and dealt with through co-creational processes to ensure progression and eventually successful implementation. Finally, the results will be presented in Fig. 1, showing the development through the four-year implementation period.

### Pre-implementation preparations

#### Involving key actors

During the planning process, the municipal top managements appointed a local project manager and involved the healthcare services and the vendors. However, contrary to internal guidelines, the municipal IT management staff were not involved until the formal decision-making was finished, which caused a series of problems and slowed down the implementation.

The municipalities anticipated small-scale projects affecting the night-shift staff and the residents being monitored. “*It seemed manageable. Just at night. Few people*” (Project manager)*.* However, the implementation involved large parts of the organisation. The IT support service and management dealt with the vendors as the monitoring technology was installed into existing IT systems and infrastructure. The healthcare managers were responsible for developing new routines, roles and responsibilities. The care providers who worked day and evening shifts had to check and prepare the technology for the night. Janitors, cleaning staff and substitute personnel needed information and tailored training to accommodate the technology into their routines. The initial failure to recognise actor complexity was followed by a consecutive involvement of all involved actors and groups over time, which facilitated the implementation.

#### Exploring system risks and compatibility

Initial risk assessments of the technology and patient safety were missing in seven of the eight municipalities. As the IT management was not involved from the very beginning, in-depth explorations of compatibility between existing and new technology did not take place until instability and errors occurred, which compromised the residents’ security and caused frustration among care providers. Instability and errors occurred in the functionality of the digital monitoring technology, but more so in the municipal system’s infrastructure and deliveries from third-party suppliers, and because of building constructions obstructing digital signal transmissions. As a quality measure, the old systems and routines operated in parallel to the new monitoring technology until stability was ensured.

#### Allocating resources

At the macro level of the healthcare system, the political expectation was that implementing digital technologies would save time and resources and be a part of the long-term solution for future resource problems. Potential cost reductions motivated the meso level of the municipal top management to initiate this implementation and was also evident at the micro level of the nursing home management. However, healthcare and IT management and staff had a dual role during the implementation because they needed to run the services as usual and simultaneously contribute to the implementation, which required extra effort and increased resources. Allocating sufficient time and resources across roles and professions for workshops and other implementation strategies proved to be a facilitator.

#### Defining roles and responsibilities

Identification and redistribution of roles and responsibilities emerged as a necessary facilitator early on and most profoundly between the IT and healthcare services. Contrary to the beliefs of the healthcare management, some IT services had not been delegated the responsibility for specific healthcare-related IT systems and support. “*The professional responsibility for applications and development related to specific needs of the municipal service sectors is delegated to the respective [healthcare] service, i.e. to a line function*” (IT manager). This was not considered by municipal top management during planning and strongly affected the co-operative climate during the early phase.

#### Maintaining leadership involvement

The mangers’ priority was operating the 24/7 healthcare service. They anticipated their own involvement to be a success factor for the implementation, but were insufficiently prepared and unable to make implementation a priority.

*I have chosen not to go into details, because I have felt that I do not … If this is something that we really want to get into, with motivated staff, I should probably get involved at some point* (Healthcare manager).

This mismatch resulted in a misalignment between the authorial structure of the organisation and the decision design architecture for the implementation, which repeatedly impeded their ability to solve unforeseen challenges.

### Implementation strategy

#### Preparing for co-creation

Unpreparedness for co-creation, which was a new way of working for most of the actors, represented a barrier during the early phase. The vendors faced a great variety of municipal practices and routines; therefore, there were no fixed solutions for their deliveries. The vendors needed the care providers’ user experience and co-operation to meet the needs of the care services and the co-operation of each municipal IT service to make the technology work in the specific settings. As the project started, the healthcare managers and staff expected the vendors to provide a tailored technology including a toolkit of new routines. When this proved not to be the case, it took time and effort for the actors to understand the concept of co-creation and contribute in a meaningful way.

#### Recognising differences between professional cultures

Cultural differences were experienced by healthcare managers and staff on the one hand and the vendors and IT staff on the other. Initially, representatives of all parties understood the basic problem to be a lack of insight or competence of the other parties. The care providers reported that neither the vendors nor IT staff understood what care for persons with dementia required. Similarly, a vendor stated that the care providers lacked interest in building their technological skills and competence. The project managers were caught in the middle and constantly needed to explain and enforce the implementation: “*There is just a ... how shall I put it … a persistent feeling that I need to translate all the time*” (Project manager)*.* Recognising these differences facilitated bridging the gap between the two cultures.

#### Facilitating dialogue and translation between professional cultures

Regular workshops were part of the innovation strategy and proved to be the prominent arena for dialogue between the two cultures (Table 2), which steadily supported progression in the implementation. The workshops became meeting places for sharing knowledge, experiences and material, as well as for common development of knowledge, new routines and distribution of responsibilities. They also led to formation of informal groups and networks such as “fast-working, self-dissolving task-teams”, where actors joined forces across organisational boundaries to solve a common problem. The team dissolved once it had identified and proposed a solution.

**Table 2:** Overview of the timing, themes, topics for co-creation activities and workshop participants in the Digital Night Surveillance Project

Date	Theme	Topics for co-creation	Participants				
			HCP	IT	T	R	Other
Nov 2014	Service innovation	Visualising the night service before and after implementation	23	3	5	6	7
		Stakeholder mapping					
		Stakeholders’ responsibilities					
		Communication between shifts					
		Need specification of new routines					
		Potential new user groups and services					
Feb 2015	Communication	Individual communication Organisational communication	27	3	4	5	1
		Communication strategies for implementation (media, public, politicians, relatives, patients, employees)					
May 2015	Service design	Future recall	28	4	4	7	5
		Patient journey, touchpoints, interactions and user experiences					
		Development of new routines					
Sept 2015	Information security and privacy	Technology improvements	10	4	2	7	2
		Requirements to infrastructure					
		Legal and regulatory issues					
Nov 2015	Routines, documentation and technology	Documentation into existing systems	21	2	4	5	
		Optimising routine descriptions, work lists, check lists. Etc					
		Optimising the technology					
		Routines and responsibilities for support					
		Patient privacy and safety					
April 2016	Service innovations and ethics	Practical familiarisation with tools in the national roadmap for welfare technology implementation and service design	17	2	3	5	2
		Ethical dilemmas when implementing monitoring technology in dementia care					
Sept 2016^a^	Implementation issues in digital surveillance technology	From pilot-testing to continuation	20	2	12	7	16
		Best practices, Norway and Sweden					
		Management challenges					
		Involvement of IT services					
		Developing clinical expertise					
		Benefit realisation					
May 2017	Results and dissemination	Final seminar	46	2	5	11	7
		Summing up experiences					
		Presenting research and project reports					

Abbreviations: *HCP* Healthcare Professionals, *IT* Information Technologists, *R* Researchers, *T* Technologists. ^a^Workshop located in Sweden

#### Establishing a team of champions

The project managers recruited care providers on the night shift and professional practice advisors as superusers of the monitoring technology. In co-operation with the vendors, they formed local implementation teams that provided technological support and training to colleagues and filled the role of implementation champions. Late appointment of superusers proved to be a barrier. In the middle phase, the teams were reorganised as a quality measure, which ensured that one superuser always was on duty to provide support around the clock. Coaching colleagues had a bonding effect between different shifts and professions, and promoted the implementation: “*I think it ties us together across professional groups*” (Night-shift nurse). Through their team efforts, they explored the other actors and reinforced the principles of co-creation to buffer frustrations and solve problems. As the mangers did not involve themselves much, the implementation teams supported each other through the consortium.

### Technology stability and usability

#### Improving reliability

Reliability of the technology was crucial to the care providers. IT infrastructure and mobile network instability was the major and persistent technological barrier: “*We had data problems from day one. That was our biggest challenge*” (Healthcare manager)*.* Some challenges were resolved, such as re-negotiation of agreements between municipalities and mobile operators to ensure priority of SMS at night, but infrastructure instability remained a challenge until the end of the implementation. In contrast, the instability in the monitoring technology portal and integrated sensor technologies was solved during the early and middle phases of the implementation.

#### Problem-solving readiness

Rapid problem solving and quick deliveries were essential to the progress of the implementation. Immediacy was hard to achieve during the early phase because of the complex distribution of responsibilities and the multitude of suppliers. Whereas the healthcare services purchased the portal directly from the vendors, third-party suppliers delivered sensor technology. The vendors and subcontractors were responsible for installing the technology. The municipal IT service and their third-party suppliers were responsible for the existing infrastructure, such as Internet and mobile networks. The IT service purchased and installed smartphones and computers, or had subcontractors doing the installation. Installation of the monitoring technology in residents’ rooms required tight coordination between the care service and all other parties. Equipment that did not work, was not delivered or installed in time caused numerous delays during the early phase, compromising safe practice as well as efficient training.

*Suddenly they* [the vendors] *were there, and then we did not have ... They did not come, and then they should install something, and … in a way we did not have a sufficient dialogue with those supposed to make the delivery. They were expected on a Friday, and suddenly they did not appear, and I had to address it, and they had not received the parts, and then they turned up on Monday … It has been a bit messy, really* (Project manager)*.*

To solve problems more rapidly, the vendors and local project managers established a routine of daily feedback on the functionality of the technology. As the implementation progressed, the feedback was maintained with less-frequent interactions.

#### Recognising tacit knowledge

Recognising tacit knowledge and tasks and types of work embedded in such knowledge was a facilitator for implementation. Tacit knowledge guided the care practices of observation by seeing, listening, smelling and feeling, and the responding clinical tasks, tailored to the needs and behaviour of the individual residents, varying from one night to another and following the progression of dementia and comorbidities over time. To adapt the technology to the care settings, the vendors had to identify the multitude of aspects of the work performed by night-shift care providers. It took time to unlock these complex and variable clinical practices through deep explorations, thorough observations of the actual work and broad discussions with the care providers.

#### Developing usability through co-creation

Involving night-shift care providers, vendors and the IT service in the systematic development of the usability of the technology proved to facilitate implementation. However, there were initially major barriers to overcome because the vendors assumed that their technology was intuitive, whereas most of the care providers found the technology all but easy to use. In addition to trial-and-error use of a variety of sensors, the complicated integration into the existing IT infrastructure increased frustration: “*You need to carry a whole notebook to remember things. And then there are all the new usernames and passwords*” (Night-shift nurse).

#### Maintaining iterative improvement

The development of the final technological solutions required considerable efforts over time from the vendors and care providers in each nursing home and within the consortium. These processes nurtured the development of mutual trust and a constructive dialogue, and vice versa. As adequate sensor technologies were installed and the technological stability and functionality improved during the middle phase of the implementation, the care providers could instantly assist the residents when needed and started to change their routines accordingly. “*It works much better now. There are few false alarms, and we tap directly into the smartphone and it simplifies a lot. It is just awesome*” (Professional development advisor nurse). The vendors also updated the technology in line with current regulations of data protection and privacy, during the course of the implementation project.

### Building competence & organisational learning

#### Tailoring iterative competence building across shifts and roles

Building competence proved to be important and necessary, but was hard to organise systematically because of the complexity of the healthcare service and the multitude of actors. Training sessions that were organised just before the shift started worked well for the night-shift workers, whereas sessions during the day were appropriate for other groups that needed training.

The project workshops facilitated iterative competence building, as did staff meetings and training sessions addressing specific needs of groups and shifts. Some needed repeated sessions and learned one task at a time. Mastering the new skills integrated into new routines required continuous practice over time for all involved care providers. Job rotation schemes, part-time positions and frequent use of substitute and on-call personnel proved to be barriers because the care providers were not sufficiently exposed to the technology. “*I need to practice [new skills] when I am working. We alternate between many wards. I only have two nights there during a six-week rotation*” (Night-shift nurse).

#### Focusing on skills

Initial training sessions were more theoretically oriented, but the care providers soon asked for instructions in the practical handling of the technology. “*Showing in practice how things work, not just telling and using words. Then we would not understand that much. We all need this for it to become second nature!*” (Night-shift healthcare worker). Skill acquisition started with learning the swiping hand movements and multi-touch gestures to handle the smartphone app and then combining the practical handling with operating the software commands before introducing the app into care settings. Until their skills were proficient, care providers perceived the technology as obstructing and stealing their focus from the residents: “*When it’s busy it feels silly to carry that phone around. We sometimes feel that we carry the phone more than actually dealing with residents*” (Night-shift nurse).

#### Overcoming language differences

In the early phase, language differences between technology and healthcare represented a barrier to learning, motivation and progress in the implementation process.

*We were speaking two different languages. They were talking about “the platform” and explained all these things about gadgets and cables and stuff, using words and phrases we didn’t understand* (Healthcare manager).

Through experience and co-operation, the vendors gradually adapted a language more understandable to the care providers. Similarly, project managers iteratively modified user manuals and written instructions into a more understandable format. Exchange of material within the consortium further facilitated competence building and implementation.

#### Organising for reflection

Some of the municipalities focused on ethical reflection, which proved to be a facilitator. Many informants appreciated face-to-face meetings to discuss dilemmas and practices. Not prioritising reflections proved to be a barrier in the later phases, undermining motivation and slowing down the implementation process.

*I can’t put my hand on my heart and claim that everybody is compliant. I don’t believe it, because it’s never discussed. I was hoping for a broader conversation and not just two minutes in a staff meeting to be informed that ‘it’s up and running’* (Night-shift healthcare worker).

### Service transformation & quality management

#### Managing risks

A series of unforeseen risks emerged in the care service during the early phase of implementation. Daytime personnel occasionally forgot to check on bed-exit sensors before residents went to bed, turned motion detectors towards the wall and moved residents in need of monitoring into rooms without sensor technology. “*Some staff working the day shift do not even know that there is a system, tugging on the cables without knowing what happens*” (Vendor). Likewise, the cleaning staff inattentively moved sensors or disconnected cables when cleaning the rooms. These incidents were communicated among the project managers, enabling them to learn from each other. As the implementation progressed, the care providers on all shifts and the support staff became more skilled and the new routines and practices reduced the risk of compromising patient safety.

#### Recognising concerns

For some actors, employment security was an important factor, especially during the early phase. Generally, older care providers seemed less technologically proficient and expressed more anxiety than younger care providers. Some actors feared that the technology would replace their roles. Others felt challenged and even embarrassed by their own poor technical proficiency. Most participants were waiting until they had received more training. A few were highly motivated from the very beginning and constituted an asset in the implementation. Professional obligation was identified as a motivating factor: “*I think it’s a part of my job as a nurse to take responsibility for it*” (Night-shift nurse)*.* Some actors, such as care assistants, felt professionally relieved from such an obligation. Feeling responsible was linked to whether one was working on the night shift, especially early on when the implementation still was considered a night-shift issue. Plans for dissemination of information and competence building contributed to relieve the concerns and deal with issues that arose as did the co-creation activities.

There were a few incidents of unfortunate patient–technology interactions, such as residents thinking the bed-exit sensor was causing them to wet their bed during the night. These incidents were solved by removing the sensors. The care providers focused on the safety aspects of the technology in their communication with relatives, who initially feared that the technology would replace human care. As the implementation proceeded, however, the relatives tended to ask for more technology.

#### Reviewing IT operating and service routines

The existing IT system operating routines not being in line with the needs and requirements of the healthcare services impeded the implementation. “*For us there are only bits and bytes. We don’t go into the context. That is the basis for how we operate*” (IT manager). Thus, the IT service workhours represented a barrier. In most of the municipalities, IT support was available during daytime on weekdays, whereas the monitoring technology was operated at night all year. When the system failed at night or during the weekend, the lack of immediate support put patients at risk. It also caused considerable distress among the night-shift staff who needed to find solutions and reorganise their care for the residents. The same thing happened when the monitoring system went down at night due to automatic IT updating routines, which resulted in alarms that did not get through to the care providers and no access to the systems. The consortium organised separate workshops focusing on IT to address these challenges. In addition, a couple of the IT managers stepped up and contributed to the project and steering groups for the benefit of healthcare services from municipalities with less involvement from their own IT services.

#### Making instructions and routines explicit and accessible to all

The lack of written night-shift instructions and explicit routines represented a barrier to service innovation. New routines for resident selection and for continuous assessments of residents’ needs (including discontinuation of digital monitoring) were gradually developed and documented into the administrative systems of the care services as were routines for maintenance of the equipment on a daily (e.g. charging), weekly (e.g. cleaning) or monthly basis (e.g. testing), and routines for reporting to and communicating with the vendors and the IT services. General awareness of the technology was integrated into existing routines, regularly commented on during staff meetings and during the handover between shifts. However, not all the work related to the technology could be included in checklists or written instructions. The superusers took responsibility for checking on sensors and wiring, sorting out issues with the phones and so on.

*It is a lot of itty-gritty things that you do that is not visible, you know. There are many things that you just drop by to check and…. I’m lying under the beds to check on the hose in port 1…. and you become…. You should have shared it with more people, but the night shift is only available at night* (Professional development advisor nurse).

Developing new procedures for the handover between night and day shifts was necessary because the technology required efforts from all of the shift workers to function as intended. This also affected the amount and content of information shared during the handover before and after the night shift. The new routines informed managers and colleagues about the residents’ behaviour during the night and they became more aware of the work of the night shift. “*It is more referred to the experiences of the night shift than before. You know, their observations are passed on to the doctor during rounds and so on*” (Healthcare manager).

#### Developing new roles

As new routines were developed, there was a need for new roles and responsibilities. In line with vendors’ recommendations, superusers were appointed among professional development advisors and night-shift care providers during the early phase. Specially trained staff administered alarm settings and patient data. This required deep knowledge about the individual residents and the task initially belonged to the primary contact nurses responsible for the residents. However, because they worked only on the day shift, the residents’ primary contact nurses did not know about their residents’ nocturnal behaviours. The alarm settings were suboptimal until the role of setting parameters was transferred to the night-shift care providers.

#### Realising benefits

New insights into the nocturnal behaviour of residents was a strong motivating factor that drove the implementation forward. The general assumption was that residents were in bed sleeping unless otherwise observed. The digital monitoring revealed that the night wanderers were far more awake during the night than previously known, even by the experienced night-shift care providers.

*The ones who seemingly were calm during the night are far more awake and wandering. I see that for some it’s normal to go to the bathroom or stretch their legs. And they do it much more often than I realised* (Night-shift healthcare worker).

Assisting the residents sooner resulted in less wandering and no elopements, in addition to more hours of sleep during night and less during the day. Ceasing the routinely rounds resulted in fewer entries into residents’ rooms. Some residents started to sleep through the night and others did not need sleep medication anymore. The care providers could co-operate and assist each other in new ways during the night shift. They saw all these benefits as increased quality of care, enhanced privacy for the residents and more efficient services, which motivated them through the final stage of implementation: “*I feel I can do an even better job, in a way, for now I can see things. So, for me, it [the implementation] has been merely positive*”. (Night-shift healthcare worker)*.* When asked about the main outcome of the implementation, a healthcare manager answered: “*In capital letters: SAFETY, both to residents, relatives and care providers*”*.* During a workshop in the last phase of the implementation, the participants were asked how the innovation project would be evaluated in 2030. “*Digital monitoring at night saves lives*”, a healthcare worker answered, “*It already has*”*.*

#### Upscaling gradually

Beneficial experiences triggered care providers to experiment and suggest upscaling to encompass more residents: “*They think it’s working well during the night and have asked if more residents could have it*” (Healthcare manager). Towards the end of the implementation, the monitoring technology was utilised for other indications than night wandering in all the nursing homes. The technology was found to be beneficial for residents with fall tendencies, infections and during rehabilitation following hip fracture.

#### The lifespan of the implementation project

From a temporal perspective, the implementation moved through characteristic phases as shown in Fig. 1. The municipal organisation represented by the management adopted the technology during the pre-implementation planning phase. The IT services, nursing home staff, residents and relatives adopted the technology during the early phase of implementation upon installation. Co-creating the adaptive elements of the monitoring technology system and instability in infrastructure characterised the early phase, with simultaneous resistance to and motivation for change. The main phase was characterised by practical experience and co-creation of service innovations. The safe and new monitoring practice, skilled care providers and realisation of benefits characterised the last phase. Following the implementation, the new, technology-based monitoring service continued. The monitoring system and the transformed services were still running in all participating nursing homes in the eight municipalities one and a half year after the completion of the implementation period (i.e., at the end of this study).

Figure 1 visualises the pre-implementation preparations, the innovation strategy and facilitators and barriers through the early, middle and late phases of the implementation.

## Discussion

This article aims to identify facilitators and barriers, and explore co-creation practices as an innovation strategy during a four-year implementation of digital monitoring technology in long-term residential care for persons with dementia who are night wanderers. The study shows that the implementation of monitoring technology in nursing homes implies radical innovation and digital transformation. The main finding – which is not previously identified – is that the complex process of digital transformation of healthcare services can be successfully facilitated by recognizing the inherent slowness of radical change and by applying co-creation methodology across roles and professions. This will be discussed in the following.

Factors that proved to facilitate the implementation when completed or impede the implementation when not completed can be categorised as: 1) Pre-implementation preparations; 2) Implementation strategy; 3) Technology stability and usability; 4) Building competence and organisational learning; and 5) Service transformation and quality management. The co-creational methodology was the most prominent facilitator and the combination of IT infrastructure instability and the reluctance of the IT support service to contribute in the co-creation of values was the most persistent barrier throughout the implementation. In combination with the project initiation followed by the pre-implementation activities, identification of three phases during implementation is in line with the five-stage model of innovation processes in organisations proposed by Rogers [33].

### The foundation for digital transformation

The implementation represented a radical and transformative innovation process in contrast to the incremental changes that all levels of management and the IT and healthcare services were prepared for. The political decision to kick-start a digital transformation of the healthcare services by implementing monitoring technology in nursing homes formed the foundation for radical innovation. The decision was in itself a strategic innovation in line with Hartley’s [30] description of long-term perspectives for restructuring responsibilities between the public care sector, the population and the private sector. The municipal managements’ initiative to enter a project with a set timeframe and a formal consortium based on the triple-helix network structure was essential. The interactions within the consortium added value to the implementation processes almost regardless of settings, participants and activities, which supports the proposition by Sørensen and Torfing [63], i.e. cross-disciplinary collaboration enriches the generation, selection and implementation of ideas, in addition to the dissemination of new practices.

### Co-producing radically new technology

The monitoring technology fulfilled the three criteria defining technological radicalness according to Dahlin and Behrens [64], i.e. novelty, uniqueness and impact on future inventions and practices, as well as the definition proposed by Chandy and Tellis [65], which includes incorporation of substantially different technology that can fulfil key customer needs better than existing products. In contrast to most technological innovations that reconfigure known technologies [66], this system was unique because it included novel IOT middleware that allowed one application to operate a variety of technologies based on different technical protocols. The care providers had experience with and could easily manage some of the sensor technologies in line with the findings of Hall et al. [4], whereas smartphones had not yet been adopted by the majority of intended users.

Norman and Verganti [32] suggested that advances in technology and change in the meaning of existing products instead of human-centred design drove radical product innovation. In this case, the vendors had entered a not-yet-existing market, which can be considered as position innovation [30] and relied on close interaction with the care providers, that took the position as the lead users [67] of the novel technology. The vendors developed deep knowledge of the services, residents and care providers through dialogue, translation and co-creation, thus minimising the potential clash described by Coiera [68] between anticipations forming the basis for software coding and the real clinical practices. These final stages of the product innovation [30] represented a paradox. The adaptability of the technology was found attractive by the care providers and managers and is considered a promoting factor in implementation [27, 69, 70], whereas the lack of completeness, which truly frustrated the care providers, is a known barrier [39] that had to be overcome. The vendors aligned their processes with those of the care services (i.e. their customers) through the co-creation activities [52] and actively planned, tested, prototyped and implemented value co-creation opportunities. The gap in competence and difference in terminology between vendors and care providers was dealt with by translation by project managers and co-creational methodology as visualisation and prototyping, in line with the recommendations by Ünsal et al. [71]. Furthermore, the vendors explored potential technological solutions and then presented a moderate number of options, which enabled the managers, project managers and care providers to make decisions, as discussed by Bratteteig and Wagner [56].

### Knowledge conversion as a mediator for service innovations

Care providers acquired skills and adopted routines that initially were perceived as incompatible and inconsistent with existing workflows. This breach is traditionally considered to be a major barrier to implementation [22, 39]. In contrast to the incremental improvements of existing practices, most of the service and process innovations represented new ways of structuring and performing tasks and responsibilities, which supports the notion of radical innovation described by Norman and Verganti [32]. The service design methodology engaged all actors during the workshops by offering them a voice in the co-creation processes and lending them an ear during the collective prioritisation of recommendations. Co-creation efforts included sharing experiences, integrating resources and learning, and resulted in mutual betterment [72]. The methodology facilitated conversion of tacit knowledge to explicit knowledge through externalisation [38], initially in the form of critiques and concerns. Little by little, the externalisation processes resulted in written material, routines and organisational learning. Organisational learning also included recognition of all the efforts and smaller tasks constantly performed without prior mentioning in written routines. This “hidden work” [73], relied on an expert level of competence because they required a trained eye and overview to be recognised and dealt with.

### Building capacities for digital transformation

The implementation brought together groups of actors with strong internal uniformity in their knowledge base, but with thick knowledge boundaries [74] between the groups, as expressed by differences in language, interpretation and motivation. Digital transformation represented a novel domain to care providers, with a prediction that learning would be more difficult and expertise would develop incrementally [35]. An array of strategies and practices promoted competence building that was radical in the sense that it elevated most care providers from expressing almost no technological knowledge to becoming experts in intuitively using the technology, which allowed them to focus on their residents. The first learning strategy was skill acquisition, which is in line with the model introduced by Dreyfus and Dreyfus [75]. The care providers’ problem-solving capability developed through the high availability of support from and interaction with the vendors. Other learning strategies included access to training, supervision, practical experience over time and collective reflections, which are known to facilitate a positive implementation climate [76]. Discontinuity in practicing newly acquired skills inhibited the development of competence, in line with the perpetual novice syndrome described by Wilson et al. [77].

Orchestration and translation was essential for the development of absorptive capacity, including communication with external organisations, between actors in the consortium and within internal subunits [35]. As HCPs, the middle managers are expected to be able to take key roles in the implementation [78], with the capability to mediate between the innovation strategy and day-to-day activities, and translate and facilitate implementation processes [77]. Their delegation of responsibility for implementation activities to project managers and professional practice advisors without delegation of authority over the nursing staff impeded the problem-solving capacity at times when it was difficult to maintain momentum during the implementation [28]. Further, it complicated the coordination between the implementation and other organisational priorities, which is a known barrier to implementation [18, 39]. Transformational leadership has been found to support innovation and readiness for change in residential aged care settings [79]. From a long-term perspective, the nursing homes lost essential leadership competence related to digital transformation upon completion of the implementation because the project managers had held temporary positions and returned to the larger municipal healthcare organisations after the implementation completed.

In a co-creation of value perspective, the interaction between service systems such as the healthcare and IT services should optimally be based on a relationship that promoted integration of mutually beneficial resources [51]. The support and services from the IT service was an integrated part of the healthcare service ecosystem [80] because the services provided by the healthcare service (i.e. their value propositions) strongly relied on deliveries from the IT service. However, in line with traditional bureaucratic silos [63], the established practices and routines of the IT services were to a large extent ignorant of the essential traits and needs of the healthcare services. During implementation, the IT services were reluctant to participate in co-creation activities and contribute to internal knowledge transfer [35], which diminished the absorptive capacity of the municipal organisations that relied on their expertise. This recurrent infrastructure instability, which is a substantial barrier in implementations of e-health applications [19, 81], impeded the implementation during all phases. The reluctance to change IT operating routines [82] and unwillingness to solve system slowdown and downtimes, which are among the major causes for negative attitudes toward health IT among nurses [44], compromised the provision of care and had a negative reinforcing effect. As most IT support staff did not actively involve themselves in the implementation, the nursing staff and vendors joined forces as a compensating measure. Consequently, the vendors filled the supporting role [83] and thus contributed to a trustful implementation climate conducive to change and characterised by benevolence.

### Trust, risk and safety across the colliding worlds of health and technology

Trust in the monitoring technology, the infrastructure, their colleagues and their own safe use of the technology was crucial for the care providers, which supports the concept that a trustful working environment contributes to the care providers’ basis for providing quality care [17] and specifically to their confidence in caring for residents with dementia [84]. Trust expresses relative security and includes the possibility for negative consequences; therefore, both trust and risk are incorporated in the decision-making [85].

The care providers’ perception of the technology having risks for residents impeded the implementation [86]. The reports of risky situations during implementation of IATs [87] emphasise how the care service managers and staff are experienced risk assessors who continuously mitigated risk with promotion of the independence of persons with dementia and reduction of the care burden. To a large degree, however, the care providers and their managers did not have the competence to assess risks created by the digital monitoring technology [88], which inhibited balancing of implementation decisions [18] so that the technology did not impose threats to patient safety. In the early phase of implementation, their low technological competence combined with poor strategies for problem solving was a striking phenomenon, which was expressed through an inability to discriminate causes of technological malfunctions. The vendors established control measures that the nursing homes adapted as the implementation proceeded. Competence building and frequent reflections fostered a collective awareness of safety issues [89] and supported the development of a safety culture [90] over time.

### The inherent slowness of radical change

A four-year implementation of any technology might seem excessive, and time and resources could probably have been saved if the planning and preparations had been more thorough. However, as the implementation represented radical innovation, a sequence of time-consuming strategies, such as competence building and establishing new routines through continuous co-creation, dialogue and translation, had to take place to enable the care providers to integrate the new monitoring service in their clinical practice and realise the benefits and co-create value with their residents. From a value perspective, the benefits are weighed towards the costs. Despite the barriers, individual and organisational interactions, resource integration and learning within and between the actors in the consortium steadily supported the endurance of the inherent slowness of radical change. The care providers became experienced innovators [27] through these efforts. Towards the end of the implementation, they took calculated risks and experimented with the technology in contrast to previous reports from implementation of monitoring technology in residential care [e.g. 16].

### Implications for practice

The key findings of this study can be summarised into three points representing the main facilitators of digital transformation and recommendations when planning innovation and implementation processes: a) involving key actors from the very start; b) organising for dialogue and co-creation throughout the implementation period; and c) planning for competence building and iterative improvement of technologies and clinical practices.

### Further research

According to this study, both the meso and micro levels of the existing healthcare ecosystems [91] will need to change to accommodate digital transformation by integrating IT competency and possibly also IT support into the healthcare organisation and service provision to benefit the value co-creation within the ecosystem and with service users. Future research into how this can be done is recommended. A quantitative study evaluating the benefits of the digital transformation, in terms of both cost savings and outcome measures related to the effectiveness of the system, is also recommended.

### Strengths and limitations

This study covers the full duration of an implementation process involving a relatively high number of participants and technical installations. The interdisciplinary research team represents a research strength with their high levels of competence within economic and organisational studies, leadership and ethics, innovation management and healthcare professional practices in psychology and nursing. The study limitations are related to the vast material, which implies that all actors affected by the implementation were not directly involved in the data collection. The residents and their families were merely passive actors in the co-creation activities of the study and the research data involving them were primarily provided by other actors. Further, more descriptive, quantitative information related to the uptake of the technology would be useful. Because this is a case study, transferability may be difficult in other situations, although the rich descriptions of the settings and participants may enable readers to determine transferability [92].

### Contributions

This study contributes to the implementation literature by identification of factors facilitating implementation of IATs in residential care services, which can be defined as radical innovation. The longitudinal nature of the study and the close research interaction with thick descriptions of the co-creation activities and facilitating factors that developed across groups and levels of actors over time [93] contribute to the literature on co-creation of healthcare services as well as of value in those settings. The digital transformation of healthcare services differs from other public sector organisations because of the complex governance and relationship to risk [94]. The study contributes to the literature on risks and safety issues, which have been poorly explored in relation to assisted living technology in the care for persons with dementia [95].

## Conclusion

The successful implementation of novel digital monitoring technology in the care services is a complex and time-consuming process, and even more so when the technology allows the care providers to adopt radically transformed clinical practices at the point of care and offer new affordances in co-creation of value with the residents and their relatives. The timeframe in combination with the co-creation activities within the consortium was a prerequisite for most of the benefits realised in this first step of digital transformation. The existing healthcare ecosystem, relying on an external service division to provide IT competence, design and support, is not sustainable. Digital transformation of the municipal healthcare services requires more advanced IT competence to be integrated directly into the provision of care and value co-creation with service users, residents, patients and their relatives.

## Data Availability

The datasets supporting the conclusions for this article consist of transcribed interviews and observations in settings with a limited number of participants. These qualitative data will not be made available for privacy reasons.

## Abbreviations

AAL: Ambient Assistive Living Technology
ERN: Etty Ragnhild Nilsen
FG: Focus Group Interview
HCP: Healthcare Professionals
HCW: Healthcare Worker
HE: Hilde Eide
IAT: Intelligent Assistive Technologies
II: Individual Interview
IOT: Internet-of-Things
IT: Information Technology
ITM: Information Technology Manager
JD: Janne Dugstad
MIDI: Measurement Instrument for Determinants of Innovation
O: Orchestrator
PC: Personal Computer
PM: Project Manager
R: Researchers
RN: Registered Nurse
SMS: Short Message Service
T: Technologists
TE: Tom Eide
